# Supplementary material for: The Small RNA Universe of Capitella teleta
Source: Front Mol Biosci. 2022 Feb 25;9:802814. doi: 10.3389/fmolb.2022.802814 (PMC8915122; doi:10.3389/fmolb.2022.802814)
Supplement: Supplementary file 1 [file DataSheet1.ZIP › Supplement/known/CAPTEscaffold_522_23011.pdf]

Provisional ID	:	CAPTEscaffold_522_23011
Score total	:	7433774.6
Score for star read(s)	:	3.9
Score for read counts	:	7433764.3
Score for mfe	:	1.8
Score for randfold	:	1.6
Score for cons. seed	:	3
Total read count	:	14581020
Mature read count	:	14579116
Loop read count	:	19
Star read count	:	1885

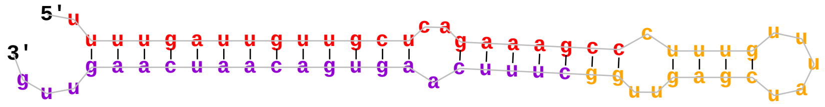

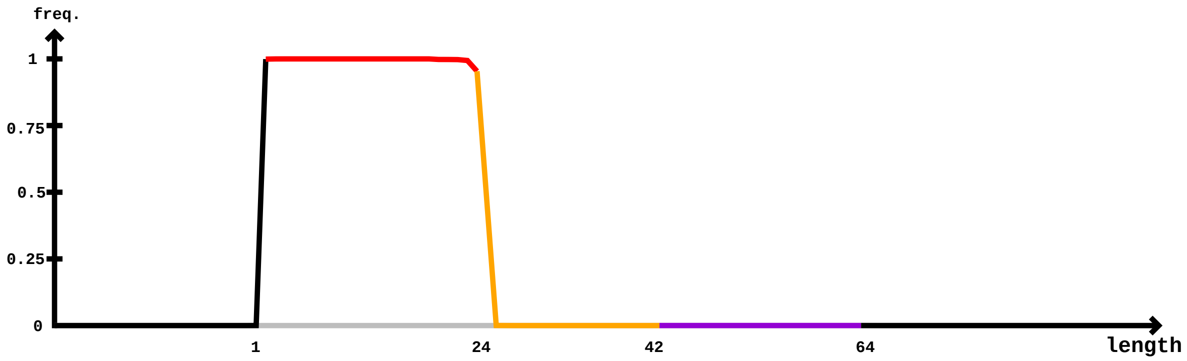


```
                        Mature                                 Star
5'- ucacuuccggcguccacaccuuuugauuguugcucagaaagcccuuuguuuaucgaguuggcuuucaagugacaaucaaguugugaaggauagccaugagcaucauugcugaa  -3'  obs
    ucacuuccggcguccacaccuuuugauuguugcucagaaagcccuuuguuuaucgaguuggcuuucaagugacaaucaaguugugaaggauagccaugagcaucauugcugaa       exp
    ucacuuccggcguccacaccuuuugauuguugcucagaaagcccuuuguuuaucgaguuggcuuucaagugacaaucaaguugugaaggauagccaugagcaucauugcugaa       known
    ........(((((((.(((..(((((((((((((..(((((((.((((.....))))..))))))).)))))))))))))..)))..)))).))).(.((((....)))).).    reads   mm   sample

    .................aUcuuuugauuguugcucagaaagc.......................................................................    9       1    seq
    .................aUcuuuugauuguugcucagaaagcc......................................................................    31      1    seq
    .................aAcuuuugauuguugcucagaaagcc......................................................................    1       1    seq
    ..................Ucuuuugauuguugcucagaa..........................................................................    1       1    seq
    ..................Ucuuuugauuguugcucagaaa.........................................................................    1       1    seq
    ..................Ucuuuugauuguugcucagaaag........................................................................    2       1    seq
    ..................Ucuuuugauuguugcucagaaagc.......................................................................    23      1    seq
    ..................Ucuuuugauuguugcucagaaagcc......................................................................    111     1    seq
    ...................Uuuuugauuguugcucaga...........................................................................    1       1    seq
    ...................cuuuugauuguugcucaga...........................................................................    6       0    seq
    ...................cuuuugauuguugcucagaa..........................................................................    1       0    seq
    ...................Auuuugauuguugcucagaaag........................................................................    1       1    seq
    ...................cuuuugauuguugcucagaaag........................................................................    2       0    seq
    ...................Auuuugauuguugcucagaaagc.......................................................................    6       1    seq
    ...................Uuuuugauuguugcucagaaagc.......................................................................    12      1    seq
    ...................cuuuugauuguugcucagaaagc.......................................................................    64      0    seq
    ...................cuuuugauugAugcucagaaagcc......................................................................    1       1    seq
    ...................Guuuugauuguugcucagaaagcc......................................................................    3       1    seq
    ...................cuuuugauuguugcucagaaagAc......................................................................    1       1    seq
    ...................cAuuugauuguugcucagaaagcc......................................................................    1       1    seq
    ...................cuuuugauuguugAucagaaagcc......................................................................    1       1    seq
    ...................cuuuugauuAuugcucagaaagcc......................................................................    1       1    seq
    ...................cuuAugauuguugcucagaaagcc......................................................................    1       1    seq
    ...................cuuuugauuguugcucagaaagcA......................................................................    3       1    seq
    ...................cuuuugauuguugcucUgaaagcc......................................................................    1       1    seq
    ...................Uuuuugauuguugcucagaaagcc......................................................................    131     1    seq
    ...................cuuuugauuguugcucagaaagcU......................................................................    4       1    seq
    ...................cuuuugauAguugcucagaaagcc......................................................................    1       1    seq
    ...................cuuuugauuguugcucagaaagcc......................................................................    539     0    seq
    ...................cuuuugauuguugcuUagaaagcc......................................................................    1       1    seq
    ...................Auuuugauuguugcucagaaagcc......................................................................    17      1    seq
```

Mature **Star**

ucacuuccggcguccacaccuuuugauuguugcucagaaagcccuuuguuuaucgaguuggcuuucaagugacaaucaaguugugaaggauagccaugagcaucauugcugaa

...................cuuuugauuguugcucagaaagccA...	5	1	seq
...................cuuuugauuguugcucagaaagccG...	1	1	seq
...................cuuuugauuguugcucagaaagccU...	371	1	seq
...................cuuuugauuguugcucagaaagccUuu...	1	1	seq
....................uuuugauugCugcucaga...	3	1	seq
....................uuuugauAguugcucaga...	11	1	seq
....................uuuugauuguugcuUaga...	18	1	seq
....................uuuugauuguugcAcaga...	9	1	seq
....................uuuugauuguuCcucaga...	1	1	seq
....................uuuugauuguugcucagC...	1	1	seq
....................uuuugauuguugcGcaga...	3	1	seq
....................uuuugUuuguugcucaga...	2	1	seq
....................uuuugauugAugcucaga...	22	1	seq
....................uuuuAauuguugcucaga...	22	1	seq
....................Cuuugauuguugcucaga...	21	1	seq
....................uuuugauuguAgcucaga...	9	1	seq
....................Nuuugauuguugcucaga...	9	1	seq
....................uuuugauuguugcucaga...	29364	0	seq
....................uuuugaCuguugcucaga...	4	1	seq
....................uuuugauuguugcuGaga...	5	1	seq
....................uuuuUauuguugcucaga...	1	1	seq
....................uuuugaAuguugcucaga...	12	1	seq
....................uuuugauuguugcucagU...	8	1	seq
....................uuuugGuuguugcucaga...	5	1	seq
....................uuuugauuguugcucagG...	35	1	seq
....................uuAugauuguugcucaga...	13	1	seq
....................uuuugauuguugcucUga...	3	1	seq
....................uuuugCuuguugcucaga...	1	1	seq
....................uuuugauuguugAucaga...	6	1	seq
....................uuuugauuguugcucGga...	1	1	seq
....................Guuugauuguugcucaga...	20	1	seq
....................uuuugauuguuAcucaga...	5	1	seq
....................uGuugauuguugcucaga...	1	1	seq
....................uuuAgauuguugcucaga...	9	1	seq
....................uuuugauuAuugcucaga...	1	1	seq
....................uuuuCauuguugcucaga...	1	1	seq
....................uuuugauuguGgcucaga...	3	1	seq
....................uuuugaGuguugcucaga...	6	1	seq
....................uNuugauuguugcucaga...	2	1	seq
....................uuuugauuguugcucaAa...	2	1	seq
....................uuuugauuguugcuAaga...	6	1	seq
....................uAuugauuguugcucaga...	13	1	seq
....................uuuugauuguugGucaga...	6	1	seq
....................uuuugauuguugcCcaga...	6	1	seq
....................uuCugauuguugcucaga...	3	1	seq
....................uuuugauuguugcucaUa...	2	1	seq
....................Auuugauuguugcucaga...	106	1	seq
....................uuuGgauuguugcucaga...	2	1	seq
....................uuuugauuCuugcucaga...	2	1	seq
....................uuuCgauuguugcucaga...	3	1	seq
....................uCuugauuguugcucaga...	7	1	seq
....................uuuugauuguugUucaga...	4	1	seq
....................uuuugauuguCgcucaga...	6	1	seq
....................uuuugauuguugcucagaa..	2274	0	seq
....................uuuAgauuguugcucagaa..	2	1	seq
....................uuuugauuguugcucagaC..	1	1	seq
....................uuuuAauuguugcucagaa..	4	1	seq
....................uuuugGuuguugcucagaa..	1	1	seq
....................uuuugauuguugcucagGa..	1	1	seq
....................uuuugauugAugcucagaa..	5	1	seq
....................uuuuUauuguugcucagaa..	1	1	seq
....................uuuugauuguugGucagaa..	1	1	seq
....................uuuugauuguugcucGgaa..	1	1	seq
....................Auuugauuguugcucagaa..	7	1	seq
....................Cuuugauuguugcucagaa..	3	1	seq
....................Nuuugauuguugcucagaa..	2	1	seq
....................uuuugauuguuAcucagaa..	1	1	seq
....................uuuugaAuguugcucagaa..	1	1	seq
....................uuuugauuguugcucagaU..	21	1	seq
....................uuuugauuguugcCcagaa..	1	1	seq

Mature **Star**

ucacuuccggcguccacaccuuuugauuguugcucagaaagcccuuuguuuaucgaguuggcuuucaagugacaaucaaguugugaaggauagccaugagcaucauugcugaa			
....................uuuugUuuguugcucagaa..	1	1	seq
....................uuuugauugGugcucagaaa...	1	1	seq
....................uNuugauuguugcucagaaa...	1	1	seq
....................uuuugauuguugcucagaaG...	4	1	seq
....................Auuugauuguugcucagaaa...	20	1	seq
....................uuuugauuguugcucaUaaa...	1	1	seq
....................uuuCgauuguugcucagaaa...	1	1	seq
....................uuuugauuguugcuGagaaa...	1	1	seq
....................uuuugauuAuugcucagaaa...	1	1	seq
....................uCuugauuguugcucagaaa...	1	1	seq
....................uuuugauuguugcucagaaa...	5315	0	seq
....................Nuuugauuguugcucagaaa...	2	1	seq
....................uuuugauuguuUcucagaaa...	1	1	seq
....................uuuugauCguugcucagaaa...	1	1	seq
....................uuuuAauuguugcucagaaa...	3	1	seq
....................uuuugauuguugcucagaGa...	1	1	seq
....................uuuugauuguugcucagaaU...	118	1	seq
....................uuuugauuguuAcucagaaa...	1	1	seq
....................uuuugauugAugcucagaaa...	1	1	seq
....................uuuugauAguugcucagaaa...	2	1	seq
....................uuuugGuuguugcucagaaa...	1	1	seq
....................uuuugauuguugcucGgaaa...	1	1	seq
....................uuuugauuguugAucagaaa...	2	1	seq
....................Cuuugauuguugcucagaaa...	5	1	seq
....................uuuugauugCugcucagaaa...	3	1	seq
....................uuuugauuguAgcucagaaa...	3	1	seq
....................uuuugaAuguugcucagaaa...	1	1	seq
....................uuuugauuguugcucagaUa...	6	1	seq
....................uuuugauuguugcucaAaaa...	1	1	seq
....................uuuugauuguugcuUagaaa...	3	1	seq
....................uuuugauuguugcucagaaC...	1	1	seq
....................uAuugauuguugcucagaaa...	1	1	seq
....................Guuugauuguugcucagaaa...	4	1	seq
....................uAuugauuguugcucagaaag..	20	1	seq
....................uuuugauuguugcucaUaaag..	4	1	seq
....................uuuGgauuguugcucagaaag..	2	1	seq
....................uuuugauuguugcucagaaaA..	484	1	seq
....................uuuugauuguugcGcagaaag..	3	1	seq
....................Auuugauuguugcucagaaag..	159	1	seq
....................uuuugauuAuugcucagaaag..	8	1	seq
....................uuuugauuguugcucagaGag..	9	1	seq
....................uuuugauuguugcuAagaaag..	16	1	seq
....................uuuugauuguugcCcagaaag..	4	1	seq
....................Nuuugauuguugcucagaaag..	8	1	seq
....................uuuugauuguuUcucagaaag..	1	1	seq
....................uuuugauuguugcucagUaag..	6	1	seq
....................uuCugauuguugcucagaaag..	4	1	seq
....................uuuugauuguugGucagaaag..	7	1	seq
....................uuuugauCguugcucagaaag..	6	1	seq
....................uuuugauuguAgcucagaaag..	14	1	seq
....................uuuuUauuguugcucagaaag..	3	1	seq
....................uuuugauuguuAcucagaaag..	9	1	seq
....................uuuAgauuguugcucagaaag..	18	1	seq
....................uuuugauuguugcucaCaaag..	2	1	seq
....................uuuugauuguugcucagaaag..	47565	0	seq
....................Cuuugauuguugcucagaaag..	23	1	seq
....................uuuugauuguugcucagaaUg..	2	1	seq
....................uuuugauugGugcucagaaag..	2	1	seq
....................uuuugauuguugUucagaaag..	5	1	seq
....................uuuugauugCugcucagaaag..	3	1	seq
....................uuuuCauuguugcucagaaag..	1	1	seq
....................uuuugauuguugcucGgaaag..	8	1	seq
....................uuuugauugAugcucagaaag..	28	1	seq
....................uuuugaAuguugcucagaaag..	13	1	seq
....................uuuugaGuguugcucagaaag..	7	1	seq
....................uNuugauuguugcucagaaag..	3	1	seq
....................uuuugauuguugcuUagaaag..	36	1	seq
....................uuuuAauuguugcucagaaag..	49	1	seq
....................uuuCgauuguugcucagaaag..	12	1	seq
....................uCuugauuguugcucagaaag..	11	1	seq

```
                    Mature                                        Star
ucacuuccggcguccacaccuuuugauuguugcucagaaagcccuuuguuuaucgaguuggcuuucaagugacaaucaaguugugaaggauagccaugagcaucauugcugaa
....................uuuugauuguugcucagaaGg........................................................................  13      1   seq
....................uuuugauuguugcucagaaaC........................................................................  5       1   seq
....................uGuugauuguugcucagaaag........................................................................  2       1   seq
....................uuuugauAguugcucagaaag........................................................................  16      1   seq
....................uuuugauuguugcucagGaag........................................................................  13      1   seq
....................uuuugauuguugcucUgaaag........................................................................  5       1   seq
....................uuuugauuguugcucCgaaag........................................................................  1       1   seq
....................uuuugGuuguugcucagaaag........................................................................  14      1   seq
....................uuuugauuUuugcucagaaag........................................................................  4       1   seq
....................uuuugauuguugAucagaaag........................................................................  9       1   seq
....................uuuugauuguugcucagaUag........................................................................  3       1   seq
....................uuuugauuguugcuGagaaag........................................................................  7       1   seq
....................uuuugauuguugcucagaaaU........................................................................  90      1   seq
....................uuuugauuguugcucaAaaag........................................................................  2       1   seq
....................uuuugaCuguugcucagaaag........................................................................  10      1   seq
....................Guuugauuguugcucagaaag........................................................................  34      1   seq
....................uuAugauuguugcucagaaag........................................................................  21      1   seq
....................uuuugauuguugcAcagaaag........................................................................  7       1   seq
....................uuGugauuguugcucagaaag........................................................................  1       1   seq
....................uuuugauuguCgcucagaaag........................................................................  5       1   seq
....................uuuugauuguGgcucagaaag........................................................................  6       1   seq
....................uuuugauuguuCcucagaaag........................................................................  2       1   seq
....................uuuugUuuguugcucagaaag........................................................................  2       1   seq
....................Guuugauuguugcucagaaagc.......................................................................  431     1   seq
....................Auuugauuguugcucagaaagc.......................................................................  1704    1   seq
....................uuCugauuguugcucagaaagc.......................................................................  69      1   seq
....................uuuugauuUuugcucagaaagc.......................................................................  23      1   seq
....................uuuugauuguugcucagGaagc.......................................................................  98      1   seq
....................uuuugauuguugcGcagaaagc.......................................................................  28      1   seq
....................uNuugauuguugcucagaaagc.......................................................................  33      1   seq
....................uuuugauuguCgcucagaaagc.......................................................................  80      1   seq
....................uuuugauuguugcucaCaaagc.......................................................................  28      1   seq
....................uuuugNuuguugcucagaaagc.......................................................................  2       1   seq
....................uuuugCuuguugcucagaaagc.......................................................................  4       1   seq
....................uuuugauuAuugcucagaaagc.......................................................................  60      1   seq
....................uuuugauugCugcucagaaagc.......................................................................  85      1   seq
....................uuuugauuguugcuAagaaagc.......................................................................  95      1   seq
....................uuAugauuguugcucagaaagc.......................................................................  291     1   seq
....................uuuugaAuguugcucagaaagc.......................................................................  199     1   seq
....................uuuuCauuguugcucagaaagc.......................................................................  25      1   seq
....................uuuugauuguugcucagaaagU.......................................................................  996     1   seq
....................uuuugauugAugcucagaaagc.......................................................................  454     1   seq
....................uuNugauuguugcucagaaagc.......................................................................  3       1   seq
....................uuuGgauuguugcucagaaagc.......................................................................  5       1   seq
....................uuuugauuguugcCcagaaagc.......................................................................  88      1   seq
....................uuuuAauuguugcucagaaagc.......................................................................  457     1   seq
....................uuuugauuguuUcucagaaagc.......................................................................  38      1   seq
....................uuuAgauuguugcucagaaagc.......................................................................  218     1   seq
....................uuuugauuguugcucagaaagc.......................................................................  575011  0   seq
....................uuuugauuguugcuUagaaagc.......................................................................  496     1   seq
....................uuuugauuguugcucagaaagG.......................................................................  191     1   seq
....................uuuugauuguugcucagaaaUc.......................................................................  38      1   seq
....................uuuugauuCuugcucagaaagc.......................................................................  30      1   seq
....................uuuugauuguugcucagaaGgc.......................................................................  96      1   seq
....................uuuugauugGugcucagaaagc.......................................................................  9       1   seq
....................uuuugauuguAgcucagaaagc.......................................................................  208     1   seq
....................uuuugauuguGgcucagaaagc.......................................................................  13      1   seq
....................Nuuugauuguugcucagaaagc.......................................................................  181     1   seq
....................uuuugauGguugcucagaaagc.......................................................................  10      1   seq
....................uuuugauuguugUucagaaagc.......................................................................  48      1   seq
....................uuuugauuguugcucagaCagc.......................................................................  3       1   seq
....................uuuugUuuguugcucagaaagc.......................................................................  54      1   seq
....................uuuugGuuguugcucagaaagc.......................................................................  107     1   seq
....................uuuugauAguugcucagaaagc.......................................................................  242     1   seq
....................uuuugauuguugcucagaaaAc.......................................................................  41      1   seq
....................uuuugauuguugcucagaaCgc.......................................................................  5       1   seq
....................uCuugauuguugcucagaaagc.......................................................................  169     1   seq
....................uuuugauuguugcucagCaagc.......................................................................  4       1   seq
....................uuuugauuguugGucagaaagc.......................................................................  49      1   seq
....................uuuugauuguugcucaAaaagc.......................................................................  120     1   seq
```

```
                    Mature                                    Star
ucacuuccggcguccacaccuuuugauuguugcucagaaagcccuuuguuuaucgaguuggcuuucaagugacaaucaaguugugaaggauagccaugagcaucauugcugaa
....................uuuugauuguuAcucagaaagc.......................................................................	62	1	seq
....................uuuugauuguuCcucagaaagc.......................................................................	23	1	seq
....................uuuugauuguugcuGagaaagc.......................................................................	46	1	seq
....................uuuugauuguugAucagaaagc.......................................................................	147	1	seq
....................uuuugauuguugcucagaUagc.......................................................................	32	1	seq
....................uuuuUauuguugcucagaaagc.......................................................................	28	1	seq
....................uuuugaGuguugcucagaaagc.......................................................................	85	1	seq
....................uAuugauuguugcucagaaagc.......................................................................	297	1	seq
....................uuuugauuguugcucaUaaagc.......................................................................	35	1	seq
....................Cuuugauuguugcucagaaagc.......................................................................	384	1	seq
....................uuuugauuguugcucagUaagc.......................................................................	36	1	seq
....................uuuugauuguugcucUgaaagc.......................................................................	59	1	seq
....................uuuugauuguugcucagaaaCc.......................................................................	20	1	seq
....................uuuugauuguugcAcagaaagc.......................................................................	72	1	seq
....................uuGugauuguugcucagaaagc.......................................................................	7	1	seq
....................uuuugauCguugcucagaaagc.......................................................................	81	1	seq
....................uGuugauuguugcucagaaagc.......................................................................	11	1	seq
....................uuuugauuguugcucaNaaagc.......................................................................	1	1	seq
....................uuuugauuguugcucagaaagA.......................................................................	2090	1	seq
....................uuuugauuguugcucCgaaagc.......................................................................	7	1	seq
....................uuuugaCuguugcucagaaagc.......................................................................	54	1	seq
....................uuuugauuguugcucagaaUgc.......................................................................	33	1	seq
....................uuuugauuguugcucGgaaagc.......................................................................	134	1	seq
....................uuuCgauuguugcucagaaagc.......................................................................	83	1	seq
....................uuuugauuguugcucagaGagc.......................................................................	122	1	seq
....................uuuugauuguugcucUgaaagcc......................................................................	749	1	seq
....................uuuugCuuguugcucagaaagcc......................................................................	55	1	seq
....................uuuugaAuguugcucagaaagcc......................................................................	2769	1	seq
....................uuuugauuguCgcucagaaagcc......................................................................	1018	1	seq
....................uuuugauuguugcucagaaagcG......................................................................	590	1	seq
....................uuuCgauuguugcucagaaagcc......................................................................	1383	1	seq
....................uuuugauuguugUucagaaagcc......................................................................	711	1	seq
....................uuuugauuguugcucagaaagcc......................................................................	7166338	0	seq
....................uuuugaCuguugcucagaaagcc......................................................................	733	1	seq
....................uuuugauuguugcucagaaaCcc......................................................................	263	1	seq
....................uNuugauuguugcucagaaagcc......................................................................	307	1	seq
....................uuuugauuguugcuAagaaagcc......................................................................	1325	1	seq
....................uuuuUauuguugcucagaaagcc......................................................................	444	1	seq
....................uuCugauuguugcucagaaagcc......................................................................	803	1	seq
....................uuuugauuUuugcucagaaagcc......................................................................	306	1	seq
....................uuuugauuguugcucagCaagcc......................................................................	70	1	seq
....................uuuugauAguugcucagaaagcc......................................................................	2822	1	seq
....................uuuugauuguuAcucagaaagcc......................................................................	1062	1	seq
....................uuuugauugAugcucagaaagcc......................................................................	5374	1	seq
....................uuuugauuguugcucCgaaagcc......................................................................	93	1	seq
....................uCuugauuguugcucagaaagcc......................................................................	1956	1	seq
....................uuuugauuguGgcucagaaagcc......................................................................	177	1	seq
....................uuuugauuguugcucagaaCgcc......................................................................	53	1	seq
....................uuuugauuguugcucagaaagNc......................................................................	1	1	seq
....................uuuugUuuguugcucagaaagcc......................................................................	617	1	seq
....................uuuugauuguugcucagaCagcc......................................................................	39	1	seq
....................uuAugauuguugcucagaaagcc......................................................................	3485	1	seq
....................uuGugauuguugcucagaaagcc......................................................................	63	1	seq
....................uuuugauuguugcCcagaaagcc......................................................................	1059	1	seq
....................uuuugauuguugcuUagaaagcc......................................................................	4825	1	seq
....................uuuugauuguugcucagaaaNcc......................................................................	3	1	seq
....................uuuugauuguugcucagaaagAc......................................................................	2044	1	seq
....................uuNugauuguugcucagaaagcc......................................................................	30	1	seq
....................uuuugauuguuCcucagaaagcc......................................................................	277	1	seq
....................uuuugauuguugGucagaaagcc......................................................................	665	1	seq
....................uuuugauuCuugcucagaaagcc......................................................................	341	1	seq
....................uuuuAauuguugcucagaaagcc......................................................................	7747	1	seq
....................Nuuugauuguugcucagaaagcc......................................................................	2383	1	seq
....................uuuugauuguugcGcagaaagcc......................................................................	340	1	seq
....................uuuGgauuguugcucagaaagcc......................................................................	197	1	seq
....................uuuugauuguugcucagaaagcN......................................................................	10	1	seq
....................uuuugauuguugcucagaaUgcc......................................................................	139	1	seq
....................uuuugauuguugcucaAaaagcc......................................................................	2369	1	seq
....................uuuugauuguugAucagaaagcc......................................................................	1448	1	seq
....................Guuugauuguugcucagaaagcc......................................................................	5725	1	seq
```

Mature **Star**

```
ucacuuccggcguccacaccuuuugauuguugcucagaaagcccuuuguuuaucgaguuggcuuucaagugacaaucaaguugugaaggauagccaugagcaucauugcugaa
....................uuuugauuguugcucagaaaUcc......................................................................   352       1   seq
....................uuuugauuguugcucagaaagUc......................................................................   627       1   seq
....................uuuugauuguugcucagaGagcc......................................................................   1815      1   seq
....................uuuugauugGugcucagaaagcc......................................................................   120       1   seq
....................uuuugauuguugcucagaUagcc......................................................................   316       1   seq
....................uuuugauuguugcucagUaagcc......................................................................   329       1   seq
....................uuuugauuguugcucagaaagcU......................................................................   10909     1   seq
....................uuuugauuAuugcucagaaagcc......................................................................   1084      1   seq
....................uuuNgauuguugcucagaaagcc......................................................................   2         1   seq
....................uuuugauuguugcucagaaaAcc......................................................................   458       1   seq
....................uuuugauuguugcucagaaagGc......................................................................   208       1   seq
....................uuuugauuguuUcucagaaagcc......................................................................   425       1   seq
....................uGuugauuguugcucagaaagcc......................................................................   150       1   seq
....................uuuugauugNugcucagaaagcc......................................................................   2         1   seq
....................uuuugauuguugcucGgaaagcc......................................................................   1526      1   seq
....................uuuugauuguugcucagaaagcA......................................................................   12128     1   seq
....................uuuugaNuguugcucagaaagcc......................................................................   1         1   seq
....................uuuuCauuguugcucagaaagcc......................................................................   322       1   seq
....................uuuugauuguugcucaNaaagcc......................................................................   2         1   seq
....................uuuugauuguugcucaUaaagcc......................................................................   376       1   seq
....................Auuugauuguugcucagaaagcc......................................................................   20577     1   seq
....................uuuugauCguugcucagaaagcc......................................................................   935       1   seq
....................uuuugauuguugcAcagaaagcc......................................................................   941       1   seq
....................Cuuugauuguugcucagaaagcc......................................................................   4628      1   seq
....................uAuugauuguugcucagaaagcc......................................................................   3564      1   seq
....................uuuAgauuguugcucagaaagcc......................................................................   2685      1   seq
....................uuuugauGguugcucagaaagcc......................................................................   98        1   seq
....................uuuugauugCugcucagaaagcc......................................................................   865       1   seq
....................uuuugauuguAgcucagaaagcc......................................................................   2415      1   seq
....................uuuugauuguugcuGagaaagcc......................................................................   628       1   seq
....................uuuugauuguugcucaCaaagcc......................................................................   324       1   seq
....................uuuugauuguugcucagGaagcc......................................................................   1399      1   seq
....................uuuugNuuguugcucagaaagcc......................................................................   26        1   seq
....................uuuugaGuguugcucagaaagcc......................................................................   967       1   seq
....................uuuugGuuguugcucagaaagcc......................................................................   1493      1   seq
....................uuuugauuguugcucagaaGgcc......................................................................   1750      1   seq
....................uuAugauuguugcucagaaagccc.....................................................................   2         1   seq
....................Nuuugauuguugcucagaaagccc.....................................................................   1         1   seq
....................Guuugauuguugcucagaaagccc.....................................................................   1         1   seq
....................uuuugauuguugcucGgaaagccc.....................................................................   1         1   seq
....................uuuugauuguugcucagaaagccG.....................................................................   69636     1   seq
....................uuuugauuguugcucagaaagccc.....................................................................   4455      0   seq
....................uuuCgauuguugcucagaaagccc.....................................................................   1         1   seq
....................uuuugauuguugcucagaaagGcc.....................................................................   3         1   seq
....................uuuugauuguugcucaAaaagccc.....................................................................   2         1   seq
....................uuuAgauuguugcucagaaagccc.....................................................................   2         1   seq
....................uuuugaCuguugcucagaaagccc.....................................................................   2         1   seq
....................uuuugauuguugcuUagaaagccc.....................................................................   2         1   seq
....................uuuugauuguugcucagaaGgccc.....................................................................   2         1   seq
....................uuuugauuguAgcucagaaagccc.....................................................................   1         1   seq
....................uuuuAauuguugcucagaaagccc.....................................................................   2         1   seq
....................uuuugauuguCgcucagaaagccc.....................................................................   1         1   seq
....................uuuugauugAugcucagaaagccc.....................................................................   6         1   seq
....................uuuugauCguugcucagaaagccc.....................................................................   2         1   seq
....................Cuuugauuguugcucagaaagccc.....................................................................   4         1   seq
....................uuuugauuguugcucagaaagccN.....................................................................   22        1   seq
....................uuuugauuguugcucagaaagcAc.....................................................................   3         1   seq
....................uuuugauuguugcucagaaagAcc.....................................................................   3         1   seq
....................uuuugauuguugcucagaaagcUc.....................................................................   4         1   seq
....................uuuugauuguugcuGagaaagccc.....................................................................   1         1   seq
....................uuuugauuguugcucaUaaagccc.....................................................................   1         1   seq
....................uAuugauuguugcucagaaagccc.....................................................................   1         1   seq
....................uuuugauuguugcucagaaagUcc.....................................................................   1         1   seq
....................uuuugauuguugAucagaaagccc.....................................................................   1         1   seq
....................Auuugauuguugcucagaaagccc.....................................................................   11        1   seq
....................uuuugauAguugcucagaaagccc.....................................................................   2         1   seq
....................uuuugauuguugcucagaaagccU.....................................................................   6448731   1   seq
....................uuuugauuguugcucagaaagccA.....................................................................   68560     1   seq
....................uuuugauuguugcucagaGagccc.....................................................................   2         1   seq
....................uuuugaGuguugcucagaaagccc.....................................................................   1         1   seq
```

```
                    Mature                                   Star
ucacuuccggcguccacaccuuuugauuguugcucagaaagcccuuuguuuaucgaguuggcuuucaagugacaaucaaguugugaaggauagccaugagcaucauugcugaa
....................uuuuCauuguugcucagaaagccc.....................................................................    1      1    seq
....................uuuugauuguugcucagaUagccc.....................................................................    1      1    seq
....................uuuugauuguugcucagaaagcccC....................................................................    2      1    seq
....................uuuugauuguugcucagaaagccUu....................................................................    2315   1    seq
....................Auuugauuguugcucagaaagcccu....................................................................    2      1    seq
....................uuuugaAuguugcucagaaagcccu....................................................................    2      1    seq
....................uuuugauuguugcucagaaagcccu....................................................................    586    0    seq
....................uuuugauuguugcucagaaagccGu....................................................................    3      1    seq
....................Nuuugauuguugcucagaaagcccu....................................................................    1      1    seq
....................uuuugauuguugcucGgaaagcccu....................................................................    1      1    seq
....................uuuugauuguAgcucagaaagcccu....................................................................    1      1    seq
....................uuuugauuguugcucagaaagcUcu....................................................................    2      1    seq
....................uuuugauuguugcucagUaagcccu....................................................................    1      1    seq
....................uuuugauuguugcucagaaagcccG....................................................................    2      1    seq
....................uuuugauuguugcucagGaagcccu....................................................................    1      1    seq
....................uuuuAauuguugcucagaaagcccu....................................................................    1      1    seq
....................uuuugauuguugGucagaaagcccu....................................................................    1      1    seq
....................Guuugauuguugcucagaaagcccu....................................................................    2      1    seq
....................uuuugauuguugcucagaaagcccA....................................................................    9      1    seq
....................uuuugauuguugcucagaaagccAu....................................................................    877    1    seq
....................uuuugauuguugcucagaaagcccuu...................................................................    1      0    seq
....................uuuugauuguugcucagaaagccAuu...................................................................    210    1    seq
....................uuuugauuguugcucagaaagccUuu...................................................................    604    1    seq
....................uuuugauuguugcucagaaagccGuu...................................................................    1      1    seq
....................uuuugauuguugcucagaaagccAuuu..................................................................    21     1    seq
....................uuuugauuguugcucagaaagccUuuu..................................................................    27     1    seq
....................uuuugauuguugcucagaaagcccuuugu................................................................    1      0    seq
....................uuuugauuguugcucagaaagcccuuuguu...............................................................    1      0    seq
.....................uuugauuguugcucagaa..........................................................................    3      0    seq
.....................uuugauuguugcucagaaa.........................................................................    4      0    seq
.....................uuugauuguugcucagaaag........................................................................    21     0    seq
.....................uuCgauuguugcucagaaagc.......................................................................    1      1    seq
.....................uuugauuguugcucagaaagA.......................................................................    1      1    seq
.....................uuugauuguugcucagaaagc.......................................................................    346    0    seq
.....................uuugauuguugcuUagaaagc.......................................................................    1      1    seq
.....................uuuCauuguugcucagaaagc.......................................................................    1      1    seq
.....................uuuUauuguugcucagaaagc.......................................................................    12     1    seq
.....................uuugauAguugcucagaaagc.......................................................................    1      1    seq
.....................uuugauuguugcuAagaaagc.......................................................................    1      1    seq
.....................uuuAauuguugcucagaaagc.......................................................................    1      1    seq
.....................uuugauuguugcucagaaGgcc......................................................................    1      1    seq
.....................Guugauuguugcucagaaagcc......................................................................    5      1    seq
.....................uuugauuguugAucagaaagcc......................................................................    2      1    seq
.....................uuugauuguugcucagaGagcc......................................................................    3      1    seq
.....................uuugauuguuAcucagaaagcc......................................................................    1      1    seq
.....................uuugauuguugcucagaaagcU......................................................................    18     1    seq
.....................Cuugauuguugcucagaaagcc......................................................................    4      1    seq
.....................Nuugauuguugcucagaaagcc......................................................................    1      1    seq
.....................uuugauuguugcucagaaUgcc......................................................................    1      1    seq
.....................uuugauuguugcucagaaagcA......................................................................    14     1    seq
.....................uuuAauuguugcucagaaagcc......................................................................    11     1    seq
.....................uuugauuguugcucagaaagcG......................................................................    1      1    seq
.....................uuuUauuguugcucagaaagcc......................................................................    103    1    seq
.....................uuugauuguugcucagaUagcc......................................................................    1      1    seq
.....................uuugauuguugcucagaaaCcc......................................................................    2      1    seq
.....................uuugauCguugcucagaaagcc......................................................................    2      1    seq
.....................uuugauuguugcucagaaagcc......................................................................    5114   0    seq
.....................uuugauuAuugcucagaaagcc......................................................................    1      1    seq
.....................uuugaCuguugcucagaaagcc......................................................................    3      1    seq
.....................uuugauAguugcucagaaagcc......................................................................    3      1    seq
.....................uuugaGuguugcucagaaagcc......................................................................    2      1    seq
.....................uuAgauuguugcucagaaagcc......................................................................    3      1    seq
.....................uuugauuguugcuAagaaagcc......................................................................    1      1    seq
.....................uuuCauuguugcucagaaagcc......................................................................    6      1    seq
.....................uAugauuguugcucagaaagcc......................................................................    2      1    seq
.....................uuugauuguugcuUagaaagcc......................................................................    2      1    seq
.....................uuugauuguAgcucagaaagcc......................................................................    3      1    seq
.....................uuugauuguCgcucagaaagcc......................................................................    1      1    seq
.....................uuugauuguugcucaAaaagcc......................................................................    3      1    seq
.....................uuugauugAugcucagaaagcc......................................................................    1      1    seq
```

Mature **Star**

ucacuuccggcguccacaccuuugauuguugcucagaaagcccuuuguuuaucgaguuggcuuucaagugacaaucaaguugugaaggauagccaugagcaucauugcugaa			
....................Auugauuguugcucagaaagcc..	11	1	seq
....................uuugaAuguugcucagaaagcc..	1	1	seq
....................uuugauuguugcucGgaaagcc..	1	1	seq
....................uuCgauuguugcucagaaagcc..	1	1	seq
....................uuugauuguugcucagaaagccA...	50	1	seq
....................uuugauuguugcucagaaagccG...	42	1	seq
....................uuugauuguugcucagaaagccU...	4071	1	seq
....................uuugauuguugcucagaaagccc...	15	0	seq
....................uuugauuguugcucagaaagccAu..	2	1	seq
....................uuugauuguugcucagaaagccUu..	1	1	seq
.....................uugauuguugcucagaaag..	2	0	seq
.....................uuUauuguugcucagaaagc...	1	1	seq
.....................uugauuguugcucagaaagc...	24	0	seq
.....................uugauuguugcucGgaaagcc..	1	1	seq
.....................uugaAuguugcucagaaagcc..	1	1	seq
.....................Augauuguugcucagaaagcc..	2	1	seq
.....................uugauuguuAcucagaaagcc..	1	1	seq
.....................uugauCguugcucagaaagcc..	1	1	seq
.....................uugauuguugcucaAaaagcc..	1	1	seq
.....................uugauuguugcucagaaagcU..	3	1	seq
.....................uugauuguugcucagaaagcc..	447	0	seq
.....................uuUauuguugcucagaaagcc..	10	1	seq
.....................uugauuguugcucagaaagccA...	7	1	seq
.....................uugauuguugcucagaaagccc...	3	0	seq
.....................uugauuguugcucagaaagccG...	6	1	seq
.....................uugauuguugcucagaaagccU...	359	1	seq
.....................uugauuguugcucagaaagccUu..	1	1	seq
......................ugauuguugcucagaaagc...	7	0	seq
......................ugauuguugcucagaaagcc..	68	0	seq
......................uAauuguugcucagaaagcc..	1	1	seq
......................Agauuguugcucagaaagcc..	1	1	seq
......................uUauuguugcucagaaagcc..	1	1	seq
......................ugauuguugcucagaaagccU...	22	1	seq
......................ugauuguugcucagaaagccA...	3	1	seq
.......................gauuguugcucagaaagc...	1	0	seq
.......................gauuguugcucagaaagcc..	18	0	seq
.......................gauuguugcucagaaagccU...	9	1	seq
........................Guuguugcucagaaagcc..	1	1	seq
........................auuguugcucagaaagcc..	26	0	seq
........................auuguugcucagaaagccU...	4	1	seq
.........................uuguugcucagaaagccU...	9	1	seq
.........................uuguugcucagaaagcUc...	1	1	seq
....cuuuguuuaucgaguugg..	16	0	seq
....cuuuguuuaucgaguuggU...	1	1	seq
....cuuuguuuaucgaguuggUuu...	2	1	seq
....Ccuuucaagugacaaucaaguug..............................	1	1	seq
....cuuucaagugacaaucaag.................................	8	0	seq
....cuuucaaguAacaaucaagu................................	1	1	seq
....cuuucaagugacaaucaagu................................	22	0	seq
....cuAucaagugacaaucaagu................................	1	1	seq
....cuuucaagugacaauUaaguu...............................	1	1	seq
....cuuucaagugacaaucaaguu...............................	168	0	seq
....cuuucaagugaUaaucaaguu...............................	1	1	seq
....cuuucaagAgacaaucaaguu...............................	1	1	seq
....cuuucaagAgacaaucaaguug..............................	1	1	seq
....cuuucaagugacaUucaaguug..............................	1	1	seq
....cuuucaagugacaaucaagAug..............................	1	1	seq
....cuuuAaagugacaaucaaguug..............................	1	1	seq
....cuuucaagugacaauUaaguug..............................	1	1	seq
....Auuucaagugacaaucaaguug..............................	2	1	seq
....cuuucaagugacaaucaaguug..............................	1455	0	seq
....Nuuucaagugacaaucaaguug..............................	3	1	seq
....cuuucaagCgacaaucaaguug..............................	1	1	seq
....cuuucaagugacaaucaaguAg..............................	1	1	seq
....cAuucaagugacaaucaaguug..............................	1	1	seq
....cuuucaagugacaaucaaguGg..............................	2	1	seq
....cuuucaagugacaaucaaguuC..............................	2	1	seq
....cuuucaagugacaaucaaguuU..............................	16	1	seq
....cuuucaGgugacaaucaaguug..............................	1	1	seq
....cuuuGaagugacaaucaaguug..............................	1	1	seq

```
                    Mature                                      Star
ucacuuccggcguccacaccuuuugauuguugcucagaaagcccuuuguuuaucgaguuggcuuucaagugacaaucaaguugugaaggauagccaugagcaucauugcugaa
.............................................................cuuucaagugacaaAcaaguug..............................   1     1     seq
.............................................................cuAucaagugacaaucaaguug..............................   1     1     seq
.............................................................cuuucaagugaUaaucaaguug..............................   1     1     seq
.............................................................cuuucaagugacaaucaaguuA..............................   4     1     seq
.............................................................Uuuucaagugacaaucaaguug..............................   3     1     seq
.............................................................cuuucaagugacaaucaaguugA.............................   2     1     seq
.............................................................cuuucaagugacaaucaaguugu.............................   15    0     seq
.............................................................cuuucaagugacaaucaaguuguU............................   1     1     seq
..............................................................uuucaagugacaaucaagu................................   3     0     seq
..............................................................uuucaagugacaaucaaguu...............................   8     0     seq
..............................................................uuucaagugacaaucaaAuug..............................   1     1     seq
..............................................................uuucaagugacaaucaaguuU..............................   4     1     seq
..............................................................uuucaagugacaaucaaguug..............................   124   0     seq
..............................................................Auucaagugacaaucaaguug..............................   1     1     seq
..............................................................uuucaaUugacaaucaaguug..............................   1     1     seq
..............................................................uuAcaagugacaaucaaguug..............................   1     1     seq
..............................................................Cuucaagugacaaucaaguug..............................   1     1     seq
..............................................................uAucaagugacaaucaaguug..............................   1     1     seq
..............................................................uuucaagugacaaucaaguugu.............................   10    0     seq
...............................................................uucaagugacaaucaaguug..............................   5     0     seq
...............................................................uucaagugacaaucaaguugu.............................   3     0     seq
```
